# Comparison of direct sequencing and amplification refractory mutation system for detecting epidermal growth factor receptor mutation in non-small-cell lung cancer patients: a systematic review and meta-analysis

**DOI:** 10.18632/oncotarget.19110

**Published:** 2017-07-08

**Authors:** Qi Feng, Zu-Yao Yang, Jia-Tong Zhang, Jin-Ling Tang

**Affiliations:** ^1^ Division of Epidemiology, JC School of Public Health and Primary Care, Faculty of Medicine, The Chinese University of Hong Kong, Hong Kong

**Keywords:** epidermal growth factor receptor, direct sequencing, amplification refractory mutation system, non-small cell lung cancer, tyrosine kinase inhibitors

## Abstract

**Background:**

Direct sequencing and amplification refractory mutation system (ARMS) are commonly used to detect epidermal growth factor receptor (*EGFR*) mutation status in patients with non-small-cell lung cancer to inform the decision-making on tyrosine kinase inhibitors treatment. This study aimed to systematically compare the two methods in terms of the rate of detected mutations and the association of detected mutations with clinical outcomes.

**Material and methods:**

PubMed, EMBASE, China National Knowledge Infrastructure (in Chinese) and Wanfang database (in Chinese) were searched to identify relevant studies. Meta-analyses of *EGFR* mutation rates, rate differences, and the associations of *EGFR* mutations with clinical outcomes of tyrosine kinase inhibitors treatment were conducted.

**Results:**

Eight hundred and sixty-six records were retrieved and 26 studies with 3282 patients were included. The pooled rate of mutations detected by ARMS (41%, 95% confidence interval (CI) 35% to 47%) was significantly higher than that by direct sequencing (28%, 95%CI 22% to 34%), with a weighted rate difference of 11% (95%CI 8% to 13%). There was a consistent trend that the associations between ARMS-detected mutations and clinical outcomes were stronger than those between direct-sequencing-detected mutations and clinical outcomes (pooled risk ratio for objective response: 5.18 *vs*. 2.25; hazard ratio for progression-free survival: 0.30 *vs*. 0.42; hazard ratio for overall survival: 0.46 *vs*. 0.54).

**Conclusions:**

More patients with *EGFR* mutations can be identified by ARMS than by direct sequencing, and those identified by ARMS seems to be able to benefit more from tyrosine kinase inhibitors than those identified by direct sequencing.

## INTRODUCTION

Epidermal growth factor receptor tyrosine kinase inhibitors (EGFR TKIs) are now the standard treatment for patients with advanced non-small cell lung cancer (NSCLC) harboring activating mutations in the *EGFR* gene [1–3]. Testing *EGFR* mutations is therefore very important for the decision-making with regard to this treatment. Two broad categories of methods, i.e. screening methods and targeted methods, are available for *EGFR* mutation testing [4]. Screening methods, such as direct sequencing, denaturing high-performance liquid chromatography and high-resolution melting analysis, detect all mutations, including novel unknown variants. Targeted methods, such as amplification refractory mutation system (ARMS), fragment length analysis and pyrosequencing, detect specific known mutations, including exon 19 deletions and exon 21 L858R point mutation that represent the majority of activating mutations of *EGFR* [4].

Direct sequencing of DNA extracted from fresh or formalin-fixed, paraffin-embedded tumor tissue is the historical standard for *EGFR* mutation testing. This method is relatively cost-effective compared with targeted methods, but it requires a mutation to be present in at least 20% of all DNA in a sample to be reliably detected [5, 6]. As the proportion of tumor cells in lung tissue samples can vary from 5% to 100% [4], and tumor tissues are often not available from advanced NSCLC for various reasons [7], in which case cytological samples such as plural effusion with low proportion of tumor cells may have to be used as alternatives, more sensitive methods for *EGFR* mutation testing are warranted. In addition, direct sequencing needs experienced operators and tends to be time-consuming and labor-intensive.

Several methods have been developed as potential alternatives to direct sequencing, each with its own limitations [4]. For example, fragment length analysis can detect insertions and deletions but not point mutations in *EGFR*; pyrosequencing requires the proportion of tumor cells in a sample to be 20% or more to maintain its accuracy [8]. Among them, the allele specific polymerase chain reaction-based method ARMS is heavily investigated, with validated, quality controlled testing kits available for use. It was employed by the landmark trial IPASS [9] to establish the predictive value of *EGFR* mutations in the EGFR TKIs treatment of advanced NSCLC, and is currently widely adopted in practice. By incorporating fluorescent probes such as TaqMan and Scorpions, ARMS can be further enhanced to analyze the results in a real-time, closed-tube format [10].

A number of studies have compared ARMS and direct sequencing in terms of the rate of *EGFR* mutations detected. While some found that the two methods yielded similar mutation rates [1, 10], others reported significantly higher mutation rate detected by ARMS than by direct sequencing [11, 12]. Whether the discrepancy across studies was caused by pure chances, different specimen types used or true difference in detecting ability between the two methods remains to be clarified. More importantly, existing studies were mainly focused on the comparison of mutation rates. Attention was rarely paid to the difference in strength of association between the *EGFR* mutations detected by different methods and clinical outcomes of EGFR TKIs treatment, which is crucial in determining the superiority of one method over the other.

We therefore conducted a systematic review with meta-analysis to address these issues. For simplicity, the comparison of ARMS with direct sequencing in this paper was focused on: (1) the ability of different methods in detecting *EGFR* mutations, as measured by mutation rate, and (2) the association of detected *EGFR* mutations with clinical outcomes of EGFR TKIs treatment. Other issues, such as logistics, requirement of expertise and costs of the methods, were not the interest of this study. The superiority of one method over the other will depend on the results of comparison in the two aspects mentioned above. Specifically, if the rate of mutations detected by ARMS is higher than by direct sequencing and the association of mutations detected by ARMS with clinical outcomes is stronger than that of mutations detected by direct sequencing, ARMS will be considered superior to direct sequencing, and vice versa. If the two methods are comparable in both aspects, they will be considered equivalent. If one of them yields higher mutation rate but the mutations detected by the other have stronger associations with clinical outcomes, then it would be hard to tell which one is superior.

## RESULTS

**Figure 1:**
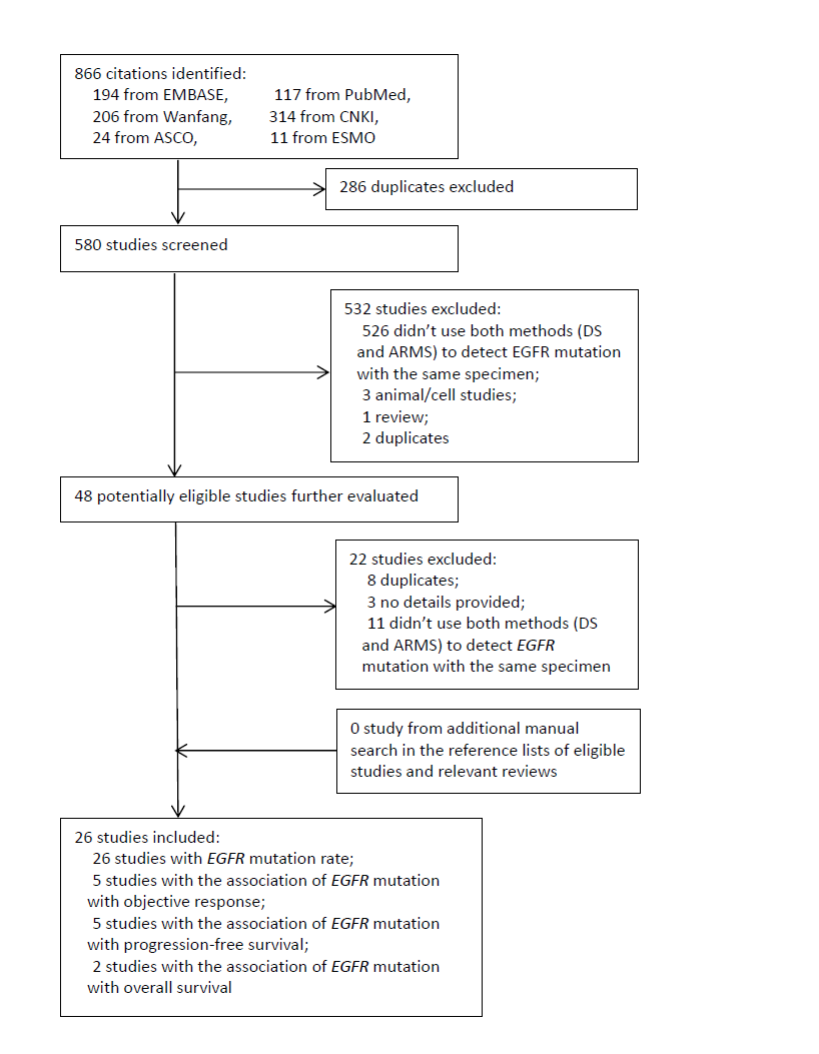
Diagram of study selection CNKI: China National Knowledge Infrastructure. ASCO: American Society of Clinical Oncology. ESMO: European Society for Medical Oncology. DS: direct sequencing. ARMS: amplification refractory mutation system. EGFR: epidermal growth factor receptor.

The results of literature search and flow of study selection are shown in Figure 1. Briefly, 26 eligible studies with 3282 NSCLC patients [1, 10–34] were identified from the 866 records initially retrieved.

### Study characteristics

Most of the included studies were from China (*n* = 20), followed by Japan (*n* = 4), South Korea (*n* = 1) and UK (*n* = 1). The sample sizes of studies ranged from 15 to 451, with a mean of 126. For *EGFR* mutation testing, 19 studies used tumor tissue samples [1, 4, 12, 14–16, 19, 20, 23, 24, 26–34], six studies cytological materials [1, 11, 13, 16, 18, 25] and three studies blood samples [18, 21, 34], with four studies using more than one type of samples [1, 16, 18, 34] and two not specifying the specimen types [17, 22]. All studies reported *EGFR* mutation rates detected by the two methods. According to the modified AHRQ quality assessment tool, the mean score was 6.2 out of 9, and eight studies (30.7%) got a score of 7 or above (Supplementary Table 1), which were considered as with high quality. The association of *EGFR* mutations with objective response, progression-free survival and overall survival was investigated by five [11, 18, 30, 32, 33] five [25, 29, 30, 32, 33] and two [25, 32] studies, respectively. All of the studies got 7 or above out of 9 scores on the Newcastle-Ottawa Scale and thus were considered as with high quality (Supplementary Table 2). The characteristics of included studies are shown in detail in Table 1.

**Table 1:** Baseline characteristics of included studies

study	Country	Test method	Sample size	Specimen type	Mutation type	Sex, male/total	Smoking status, never smoker/total	Histology, adenocarcinoma/total	Outcomes	Study quality assessment	
										AHRQ score(out of 9)	Newcastle-Ottawa score(out of 9)
Chu H 2013	China	DS vs ARMS	24	cytological	exon 19-21	16/24	8/24	20/24	mutation rate	8	NA^3^
Dou Y 2013	China	DS vs ARMS	199	tumor tissue	exon 19,21	146/199	NA	103/199	mutation rate	6	NA^3^
Ellison G 2010	UK	DS vs ARMS	197	tumor tissue	exon 18-21	NA	NA	NA	mutation rate	6	NA^3^
Goto K 2012	Japan	DS vs ARMS	135	tumor tissue, cytological	exon 18-21	NA	NA	NA	mutation rate	5	NA^3^
Horiike A 2007	Japan	DS vs ARMS	91	tumor tissue	exon 19, 21	63/94	34/94	58/94	mutation rate	6	NA^3^
Kimura H 2006	Japan	DS vs ARMS	24	cutological	exon 18-21	13/24	14/24	23/24	mutation rate, ORR	6	8
Lee D 2010	South Korea	DS vs ARMS	21	tumor tissue	exon 18, 19, 21	8/21	13/21	20/21	mutation rate	NA^2^	NA^2^
Li C 2014	China	DS vs ARMS	451	tumor tissue, cytological	exon 18-21	204/451	NA	329/406	mutation rate	7	NA^3^
Li H 2011	China	DS vs ARMS	15	NA^1^	exon 18-21	NA	NA	NA	mutation rate	NA^2^	NA^2^
Liu Y 2011	China	DS vs ARMS	50	blood, cytological	exon 19, 21	32/50	NA	50/50	mutation rate, ORR	5	8
Morinaga R 2008	Japan	DS vs ARMS	100	tumor tissue	exon 18-21	64/100	33/100	61/100	mutation rate	7	NA^3^
Qian X 2015	China	DS vs ARMS	131	tunor	exon 18-21	NA	NA	NA	mutation rate	7	NA^3^
Qin L 2011	China	DS vs ARMS	73	blood	exon 19, 21	33/73	48/73	73/73	mutation rate	6	NA^3^
Shujie A 2014	China	DS vs ARMS	154	NA^1^	exon 18-21	84/154	NA	121/154	mutation rate	6	NA^3^
Wang J 2012	China	DS vs ARMS	45	tumor tissue	exon 18-21	21/45	NA	28/45	mutation rate	7	NA^3^
Wang S 2012	China	DS vs ARMS	37	tumor tissue	exon 18-21	127/241	126/241	213/241	mutation rate	7	NA^3^
Wang Z 2014	China	DS vs ARMS	180	cytological	exon 18-21	109/180	NA	177/180	mutation rate, PFS, OS	7	8
Xu H 2014	China	DS vs ARMS	182	tumor tissue	exon 18-21	126/220	NA	183/220	mutation rate	8	NA^3^
Zhang J 2008	China	DS vs ARMS	82	tumor tissue	exon 18-21	48/82	NA	39/82	mutation rate	6	NA^3^
Zhang X 2013	China	DS vs ARMS	420	tumor tissue	NA	NA	NA	420/420	mutation rate	NA^2^	NA^2^
Zhao J 2011	China	DS vs ARMS	31	tumor tissue,	exon 19, 21	NA	NA	NA	mutation rate	5	NA^3^
Zhao J 2013	China	DS vs ARMS	168	tumor tissue	exon 18-21	96/168	109/168	125/168	mutation rate, ORR, PFS	6	9
Zhao J 2014	China	DS vs ARMS	124	tumor tissue	exon 18-21	43/124	97/124	105/124	mutation rate, PFS	4	8
Zhou Q 2011	China	DS vs ARMS	100	tumor tissue	exon 18-21	49/100	77/100	93/100	mutation rate, ORR, PFS, OS	6	8
Zhou S 2014	China	DS vs ARMS	158	tumor tissue	exon 19, 21	86/158	88/158	101/158	mutation rate, ORR, PFS	5	8
Zou M 2013	China	DS vs ARMS	90	tumor tissue, blood	exon 19, 21	55/90	59/90	57/90	mutation rate	6	NA^3^

DS: direct sequencing; ARMS: amplification refractory mutation system; ORR: objective response rate; OS: overall survival; PFS: progression-free survival; NA^1^: the study did not provide sufficient information on specimen types; NA^2^: conference abstract without sufficient information for quality assessment; NA^3^: cross-sectional studies, inapplicable for cohort study quality assessment.

### *EGFR* mutation rate

**Figure 2:**
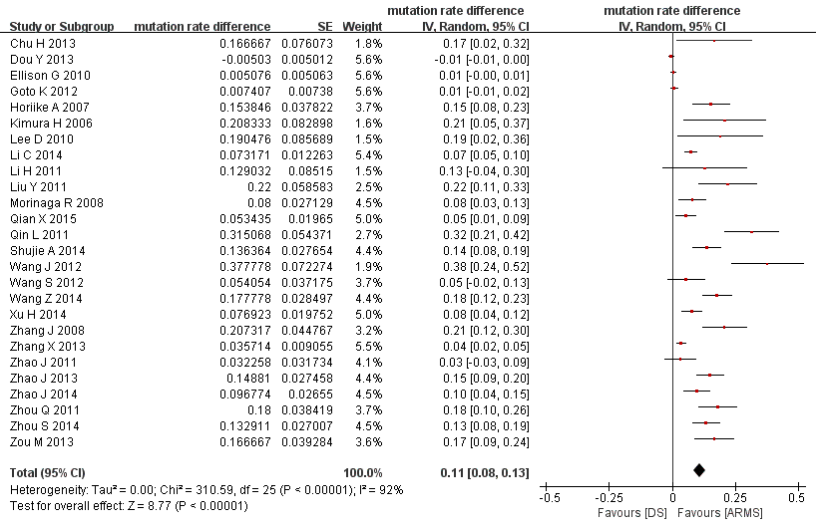
Meta-analysis of mutation rate differences between ARMS and direct sequencing DS: direct sequencing. ARMS: amplification refractory mutation system.

The rates of *EGFR* mutations detected by ARMS ranged from 9% to 69%, with a pooled mutation rate of 41% (95% confidence interval (CI) 35%-48%; heterogeneity test: *P* < 0.001, I^2^ = 92%). The mutation rates detected by direct sequencing ranged from 4% to 60%, with a pooled mutation rate of 28% (95%CI 23%-35%; heterogeneity test: *P* < 0.001, I^2^ = 93%). The within-study difference in mutation rate between ARMS and direct sequencing ranged from -1% to 38%, with a pooled rate difference of 11% (95%CI 8% to 13%; heterogeneity test: *p* < 0.001, I^2^ = 92%; Figure 2), which indicated that the mutation rate by ARMS was significantly higher than by direct sequencing. Subgroup analyses were conducted to investigate the significant heterogeneity among studies. The results (Table 2) showed that mutation rate differences were significantly larger in Asian than non-Asian population, in the studies with big sample sizes than those with small, and in cytological and blood samples than in tumor tissue, while not influenced much by study quality. Meta-regression analysis showed that the four factors contributed 27.6% to the overall heterogeneity, and sample size was the only factor that reached statistical significance (*p* = 0.008).

**Table 2:** Meta-analyses of mutation rates detected by ARMS and direct sequencing and their difference

Group/Subgroup (number of studies)	ARMS mutation rate(95%CI) (a)	DS mutation rate(95%CI) (b)	Mutation rate difference(95%CI) (a-b)	Test for difference (a-b) across subgroups
Overall (26)	0.41(0.35,0.48)	0.28(0.23,0.35)	0.11(0.08,0.13)	
**Ethnicity**				*p* < 0.001
UK (1)	0.09(0.06,0.14)	0.08(0.05,0.13)	0.01(0.00,0.02)	
Asian (25)	0.43(0.38,0.49)	0.3(0.24,0.36)	0.12(0.09,014)	
**Sample size**				*p* < 0.001
Big (12)	0.36(0.27,0.44)	0.27(0.19,0.37)	0.07(0.04,0.10)	
Small (14)	0.48(0.39,0.57)	0.3(0.21,0.40)	0.17(0.12,0.22)	
**Study quality**				*p* = 0.93
High (8)	0.45(0.38, 0.53)	0.33(0.26,0.40)	0.11(0.07,0.15)	
Low (18)	0.39(0.31, 0.49)	0.26(0.19,0.36)	0.11(0.08,0.14)	
**Specimen type**				*p* = 0.02
Tumor tissue (19)	0.42(0.34,0.50)	0.31(0.24,0.39)	0.08(0.05,0.10)	
Cytological sample (6)	0.5(0.38,0.61)	0.36(0.24,0.50)	0.16(0.07,0.24)	
Blood (3)	0.28(0.16,0.44)	0.05(0.03,0.10)	0.24(0.11,0.36)	

ARMS: amplification refractory mutation system.

DS: direct sequencing.

Funnel plots for detecting potential publication bias were not constructed because of the substantial heterogeneity between included studies [35, 36].

### Association of *EGFR* mutation status and clinical outcomes

**Figure 3:**
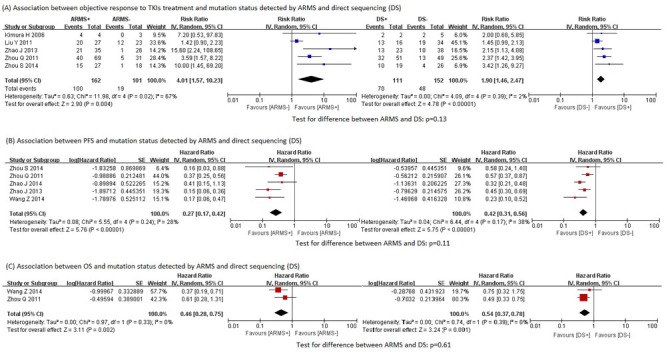
Associations between clinical outcomes and *EGFR* mutation status detected by ARMS and direct sequencing **A**. Associations between objective response to TKIs treatment and *EGFR* mutation status detected by ARMS and direct sequencing. **B**. Associations between PFS and *EGFR* mutation status detected by ARMS and direct sequencing. **C**. Association between OS and *EGFR* mutation status detected by ARMS and direct sequencing. ARMS: amplification refractory mutation system. DS: direct sequencing. EGFR: epidermal growth factor receptor. PFS: progression-free survival. OS: overall survival.

As expected, the *EGFR* mutations detected were significantly associated with all the three clinical outcomes of EGFR TKIs treatment, regardless of the testing method used (Figure 3). The association of mutations by ARMS with clinical outcomes was consistently stronger than that of mutations by direct sequencing with clinical outcomes (RR for objective response rate: 4.01 vs 1.89, test for difference *p* = 0.13; HR for progression-free survival: 0.27 vs 0.42, test for difference *p* = 0.11; HR for overall survival: 0.46 vs 0.54, test for difference *p* = 0.61), although the difference between the two methods were not always statistically significant, possibly due to limited number of studies. For the same reason, subgroup analyses and funnel plots were not performed [37]. Sensitivity analyses according to study quality were also not conducted, because all studies in the meta-analyses were of relatively high quality.

## DISCUSSION

This systematic review included 26 studies with 3282 NSCLC patients and compared direct sequencing and ARMS in terms of the rate of mutations detected and the associations of detected mutations with clinical outcomes of EGFR TKIs treatment.

The pooled rate of mutations detected by ARMS was higher than that by direct sequencing and the difference was statistically significant. This finding could be a result of the differential sensitivity of the two methods [18]. Generally speaking, direct sequencing requires the mutant tumor cells to take up 20% or more of total tumor cells for detection [5, 6], while ARMS can detect mutations with much lower concentration (as low as 1%) because it selectively amplifies mutation sequences that are identified with specific probes [10, 33]. Thus, patients with *EGFR* mutations are more likely to be identified by ARMS than by direct sequencing. For example, in Hideharu's [11] and Liu's [18] studies, ARMS identified *EGFR* mutant patients that were deemed “negative” by direct sequencing, while no patients “negative” on ARMS was identified as mutants by direct sequencing. People may argue that ARMS is disadvantaged by the fact that it can detect only known mutants while direct sequencing can detect both known and unknown. But actually, common mutations (such as the exon 19 *deletion* and the exon 21 *L858R* point mutation) and many uncommon subtypes (such as exon 18 *G719X*, exon 20 *Ins*, exon 20 *T790M*), which comprise over 95% of all *EGFR* mutations [38], can be detected by both methods. Thus, this issue is unlikely to constitute a major limitation of ARMS.

The ARMS-detected *EGFR* mutations seemed to have stronger association with clinical outcomes of EGFR TKIs treatment than did direct-sequencing-detected mutations, although the differences between the two were not always statistically significant possibly due to the limited number of studies available.

One potential explanation for this finding might have to do with the proportion of key mutation subtypes that are highly related to the efficacy of TKIs. *EGFR* mutations contain multiple subtypes occurring within the exon 18/19/20/21, and they respond to TKIs differentially [38, 39]. Previous studies indicated that mutations in exon 18/19/21 are sensitive but those in exon 20 are resistant to TKIs [38]. Since direct sequencing could identify more other unknown subtypes than ARMS, the proportion of key common mutations out of all detected mutations was supposed to be lower for direct sequencing than for ARMS, leading to “dilution” of the association between direct-sequencing-detected mutations as a whole and the efficacy of TKIs, in contrast to the situation of ARMS. However, after revisiting all included studies, we found that the proportion of sensitive mutations (exon 18/19/21 mutations) out of all were around 98% for both ARMS and direct sequencing. Thus, this explanation is not supported by data from empirical studies.

Another potential mechanism is that direct sequencing has a higher false negative misclassification rate than does ARMS [33]. False negative patients are categorized as non-mutant ones, but actually they benefit from EGFR TKIs. Thus, a higher false negative rate means that the association between mutation status and treatment efficacy is diluted to a larger extent. This seems to be a more plausible explanation for our findings.

So far, we can see that ARMS is better than direct sequencing in terms of both the *EGFR* mutation rate and the ability of detected mutations in predicting efficacy of EGFR TKIs. However, based on this comparison alone, it is still not straightforward whether ARMS is more favorable than direct sequencing in clinical practice, as other factors should be taken into consideration as well when deciding which method to use. Gillian et al [4] proposed a framework for choosing an appropriate detection method, which consists of three main components, i.e. sample type, relevant expertise and equipment, and whether detection of known mutations only is sufficient.

The results of our subgroup analyses showed that the mutation rates detected by ARMS (range: 28% to 50%) varied less than those by direct sequencing (range: 5% to 36%) when different types of biological samples were used. This finding has important implications for clinical practice. Since tumor tissues are often difficult to obtain and frequently not available from advanced patients, cytological materials and blood samples have been suggested to be used as the substitutes. For example, the College of American Pathologists, the International Association for the Study of Lung Cancer and the Association for Molecular Pathology have recommended the use of cytological materials in *EGFR* mutation testing since October 2013 [40]. Given its better performance in these types of samples, ARMS seems to have more advantages than direct sequencing in clinical practice.

This study has several limitations. Firstly, the small number of included studies that evaluated the association of detected mutation status with clinical outcomes prevented us from drawing a firm conclusion on the superiority of predictive value of mutations detected by different methods. Secondly, for difference in mutation rates between the two methods, only about one third of the studies were of high quality. However, subgroup analysis showed that the results from high quality studies were consistent with those form low quality ones. Thus, we argue that this issue is unlikely to be a major problem. Thirdly, substantial heterogeneity was observed in the meta-analysis of mutation rate differences, but pre-planned subgroup and meta-regression analyses failed to identify the major source of the heterogeneity, which undermined the validity of our findings. Fourthly, test for publication bias was not performed, due to either the significant heterogeneity or the small number of included studies. Lastly, as most included studies were conducted in Asian population, caution should be taken in generalizing the results to Western population.

In conclusion, more NSCLC patients with *EGFR* mutations can be identified by ARMS than by direct sequencing, and those identified by ARMS seems to be able to benefit more from tyrosine kinase inhibitors than do those identified by direct sequencing. In terms of the technical performance alone, ARMS represents a valid alternative to direct sequencing for testing *EGFR* mutations.

## MATERIALS AND METHODS

### Search strategy

We systematically searched PubMed, EMBASE, China National Knowledge Infrastructure (CNKI, in Chinese) and Wanfang database (in Chinese) through May 2015 to identify relevant studies. The combination of the following three groups of terms (or their Chinese counterparts) were used for the search: (1) “lung” and “pulmonary”; (2) “cancer”, “carcinoma”, “adenocarcinoma”, and “tumor”; and (3) “sequenc*”, “amplification refractory mutation system”, “ARMS”, “allele-specific polymerase chain reaction ”, “allele-specific PCR”, “PASA”, and “ASP”. The search terms were limited to title/abstract and the studies were limited to “human” where possible. The abstracts of relevant conferences of American Society of Clinical Oncology, European Society for Medical Oncology European Lung Cancer Conference were also searched. The reference lists of eligible studies and relevant reviews were manually checked for additional studies.

### Study selection

One reviewer (JTZ) screened the titles and abstracts of the retrieved records to judge their relevance, with the potentially eligible studies subject to full text examination. This process was double-checked by a second reviewer (QF). The disagreements between the two, if any, were resolved by discussion or by consulting a third reviewer (ZYY). To be eligible for inclusion into this meta-analysis, original studies had to meet the following criteria: (1) Patients were diagnosed with advanced NSCLC; (2) Pre-treatment *EGFR* mutation status of some or all patients was tested by both ARMS and direct sequencing; (3) For each patient tested, the samples used for ARMS and directing sequencing were from the same source, e.g. tumor tissue; (4) Mutation rate detected by each method was reported or could be calculated from reported data; and (5) (optional) the associations of *EGFR* mutation status detected by different methods with clinical outcomes of EGFR TKIs treatment were reported. If more than one record were identified for an eligible study, only the most complete one was included.

### Data extraction

The following information was extracted by one reviewer(JLZ) from eligible studies using a pre-designed data extraction form and double-checked by a second reviewer (QF): (1) bibliographic information, such as first author's name, year of publication, study country, and sample size; (2) patients’ characteristics, such as the proportions of male, smokers and adenocarcinoma; (3) results of *EGFR* mutation testing, such as the number of patients with *EGFR* mutations detected by ARMS and direct sequencing, respectively, the types of specimen used; (4) clinical outcomes of EGFR TKIs treatment, including objective response rate, progression-free survival and overall survival, and their corresponding risk ratios (RRs) or hazard ratios (HRs); and (5) other information needed for study quality assessment (see below).

### Quality assessment

The data on *EGFR* mutation rate were obtained from cross-sectional studies or the baseline data of cohort studies, which was similar to cross-sectional studies in nature. We employed an 11-item tool recommended by the Agency for Healthcare Research and Quality (AHRQ) to assess the quality of those data, which had been employed by previous systematic reviews [41, 42]. As the item 8 for confounding and item 11 for follow-up of the tool were not applicable in the quality assessment of prevalence study, only nine items remained in our assessment, with 1 score assigned to each item if it was satisfied and 7 or above out of 9 scores regarded as high quality.

The data on association of *EGFR* mutations with clinical outcomes were obtained from cohort studies, for which the Newcastle-Ottawa Scale was employed for quality assessment. The Newcastle-Ottawa Scale consists of three main constructs, i.e. selection (4 items), comparability (1 item) and outcome (3 items) [43]. One star was given to each item if it was met by the study being assessed, except item 5 for comparability, which could be given a maximum of 2 stars. A study with 7 or above out of 9 stars were regarded as high quality [44]. We did not assess the quality of conference abstracts, because the information needed for quality assessment were usually not available from them and their quality could turn out to be misleadingly low.

### Statistical analysis

The mutation rates detected by ARMS and direct sequencing, respectively, were combined to obtain an overall mutation rate for each method. To evaluate the difference between the two method, a mutation rate difference (ARMS minus direct sequencing) was calculated within each study and then the rate differences from all relevant studies were synthesized to obtain an overall estimates. If the difference was statistically greater than zero, then the mutation rate by ARMS was considered as higher than that by direct sequencing. To compare the predictive ability of *EGFR* mutation status detected by ARMS with that by direct sequencing, firstly the associations of *EGFR* mutation status detected by each method with clinical outcomes were meta-analyzed across studies to obtain overall estimates, and then the two overall estimates for each outcome were compared to see which one was stronger. Three clinical outcomes, i.e. objective response rate, progression-free survival and overall survival, were of our interest. The association of *EGFR* mutation status with the outcomes was measured by RR (for objective response) or HR (for progression-free survival and overall survival).

Meta-analyses were conducted with the random-effects model. Heterogeneity was assessed by the Cochran's Q test and the I^2^ statistic. A *P* value ≤ 0.10 for the Q test or an I^2^ ≥ 50% suggested the presence of substantial heterogeneity between studies. The difference in association of clinical outcomes and *EGFR* mutation status detected by two methods was examined by a method similar to the heterogeneity test. Subgroup analyses stratified by sample size, study quality, ethnicity, specimen types and meta-regression incorporating all these factors were conducted to investigate the potential sources of heterogeneity. We planned to assess publication bias by using funnel plots, but did not actually do so because of the substantial heterogeneity in the meta-analyses of *EGFR* mutation rates and the limited number (<10) of studies in the meta-analyses of RRs and HRs [35, 36]. We employed a two-tailed significance level of 0.05 for all the statistical tests except for the heterogeneity tests, for which the significance level is 0.10. Data analyses were conducted with RevMan 5.1, STATA 11.0 and Comprehensive Meta-Analysis 2.2.

### Compliance with ethical standards

Ethical approval: This article does not contain any studies with human participants or animals performed by any of the authors.

## SUPPLEMENTARY MATERIALS FIGURES AND TABLES







## Abbreviations

EGFR: epidermal growth factor receptor
TKI: tyrosine kinase inhibitor
NSCLC: non-small cell lung cancer
ARMS: amplification refractory mutation system
CI: confidence interval
RR: risk ratios
HR: hazard ratio
