# Supplementary material for: Comparison of direct sequencing and amplification refractory mutation system for detecting epidermal growth factor receptor mutation in non-small-cell lung cancer patients: a systematic review and meta-analysis
Source: Oncotarget. 2017 Jul 8;8(35):59552–62. doi: 10.18632/oncotarget.19110 (PMC5601754; doi:10.18632/oncotarget.19110)
Supplement: Supplementary file 1 [file oncotarget-08-59552-s001.pdf]

Comparison of direct sequencing and amplification refractory mutation system for detecting epidermal growth factor receptor mutation in non-small-cell lung cancer patients: a systematic review and meta-analysis

SUPPLEMENTARY MATERIALS

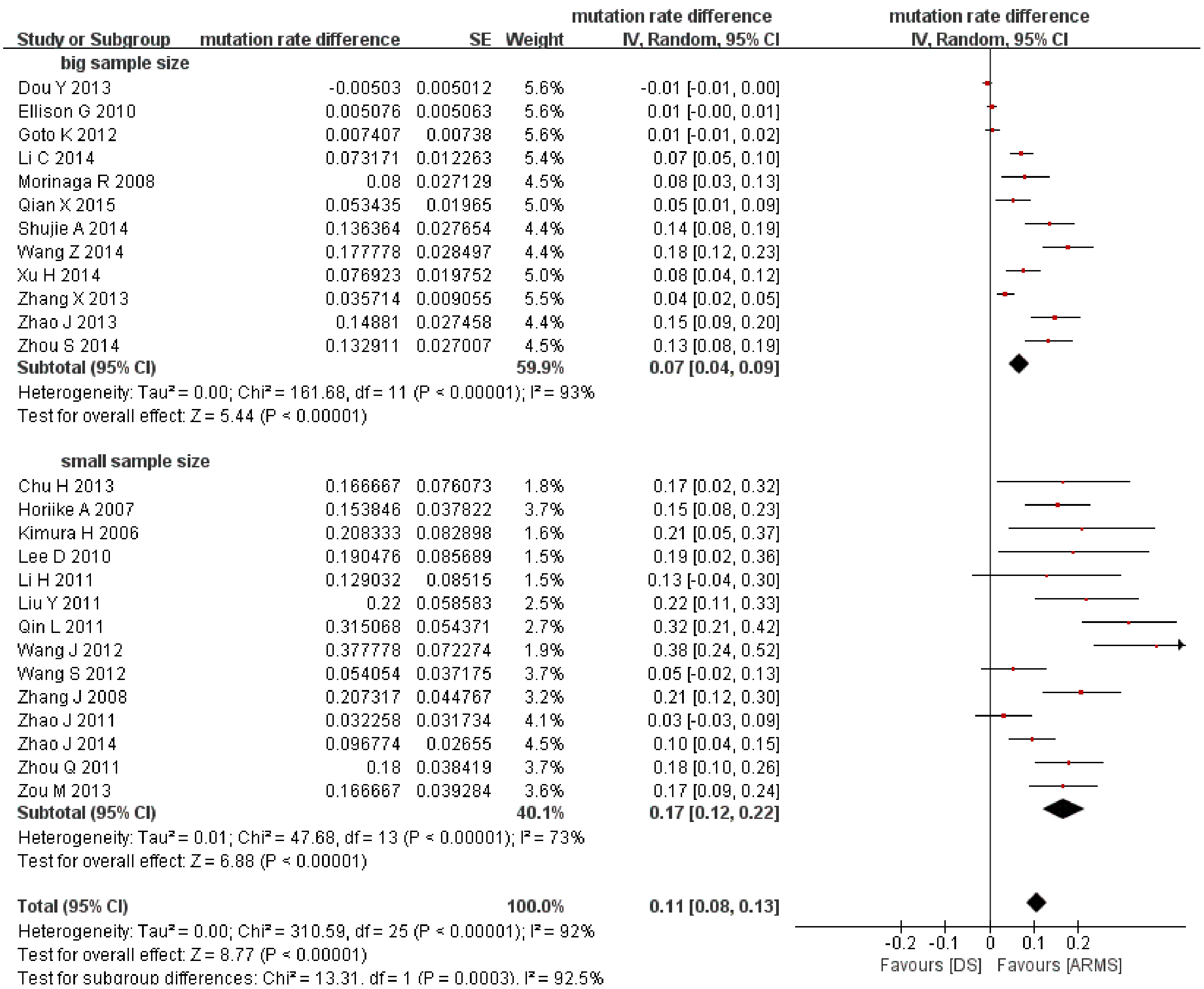

Supplementary Figure 1: Meta-analysis of mutation rate differences stratified by sample size.

Study or Subgroup	mutation rate difference	SE	Weight	mutation rate difference IV, Random, 95% CI
**high quality studies**				
Chu H 2013	0.166667	0.076073	1.8%	0.17 [0.02, 0.32]
Li C 2014	0.073171	0.012263	5.4%	0.07 [0.05, 0.10]
Morinaga R 2008	0.08	0.027129	4.5%	0.08 [0.03, 0.13]
Qian X 2015	0.053435	0.01965	5.0%	0.05 [0.01, 0.09]
Wang J 2012	0.377778	0.072274	1.9%	0.38 [0.24, 0.52]
Wang S 2012	0.054054	0.037175	3.7%	0.05 [-0.02, 0.13]
Wang Z 2014	0.177778	0.028497	4.4%	0.18 [0.12, 0.23]
Xu H 2014	0.076923	0.019752	5.0%	0.08 [0.04, 0.12]
**Subtotal (95% CI)**			**31.6%**	**0.11 [0.07, 0.15]**
Heterogeneity: Tau² = 0.00; Chi² = 32.52, df = 7 (P < 0.0001); I² = 78%				
Test for overall effect: Z = 5.18 (P < 0.00001)				
**low quality studies**				
Dou Y 2013	-0.00503	0.005012	5.6%	-0.01 [-0.01, 0.00]
Ellison G 2010	0.005076	0.005063	5.6%	0.01 [-0.00, 0.01]
Goto K 2012	0.007407	0.00738	5.6%	0.01 [-0.01, 0.02]
Horiike A 2007	0.153846	0.037822	3.7%	0.15 [0.08, 0.23]
Kimura H 2006	0.208333	0.082898	1.6%	0.21 [0.05, 0.37]
Lee D 2010	0.190476	0.085689	1.5%	0.19 [0.02, 0.36]
Li H 2011	0.129032	0.08515	1.5%	0.13 [-0.04, 0.30]
Liu Y 2011	0.22	0.058583	2.5%	0.22 [0.11, 0.33]
Qin L 2011	0.315068	0.054371	2.7%	0.32 [0.21, 0.42]
Shujie A 2014	0.136364	0.027654	4.4%	0.14 [0.08, 0.19]
Zhang J 2008	0.207317	0.044767	3.2%	0.21 [0.12, 0.30]
Zhang X 2013	0.035714	0.009055	5.5%	0.04 [0.02, 0.05]
Zhao J 2011	0.032258	0.031734	4.1%	0.03 [-0.03, 0.09]
Zhao J 2013	0.14881	0.027458	4.4%	0.15 [0.09, 0.20]
Zhao J 2014	0.096774	0.02655	4.5%	0.10 [0.04, 0.15]
Zhou Q 2011	0.18	0.038419	3.7%	0.18 [0.10, 0.26]
Zhou S 2014	0.132911	0.027007	4.5%	0.13 [0.08, 0.19]
Zou M 2013	0.166667	0.039284	3.6%	0.17 [0.09, 0.24]
**Subtotal (95% CI)**			**68.4%**	**0.11 [0.08, 0.13]**
Heterogeneity: Tau² = 0.00; Chi² = 217.06, df = 17 (P < 0.00001); I² = 92%				
Test for overall effect: Z = 7.54 (P < 0.00001)				
**Total (95% CI)**			**100.0%**	**0.11 [0.08, 0.13]**
Heterogeneity: Tau² = 0.00; Chi² = 310.59, df = 25 (P < 0.00001); I² = 92%				
Test for overall effect: Z = 8.77 (P < 0.00001)				
Test for subgroup differences: Chi² = 0.00, df = 1 (P = 0.99), I² = 0%				

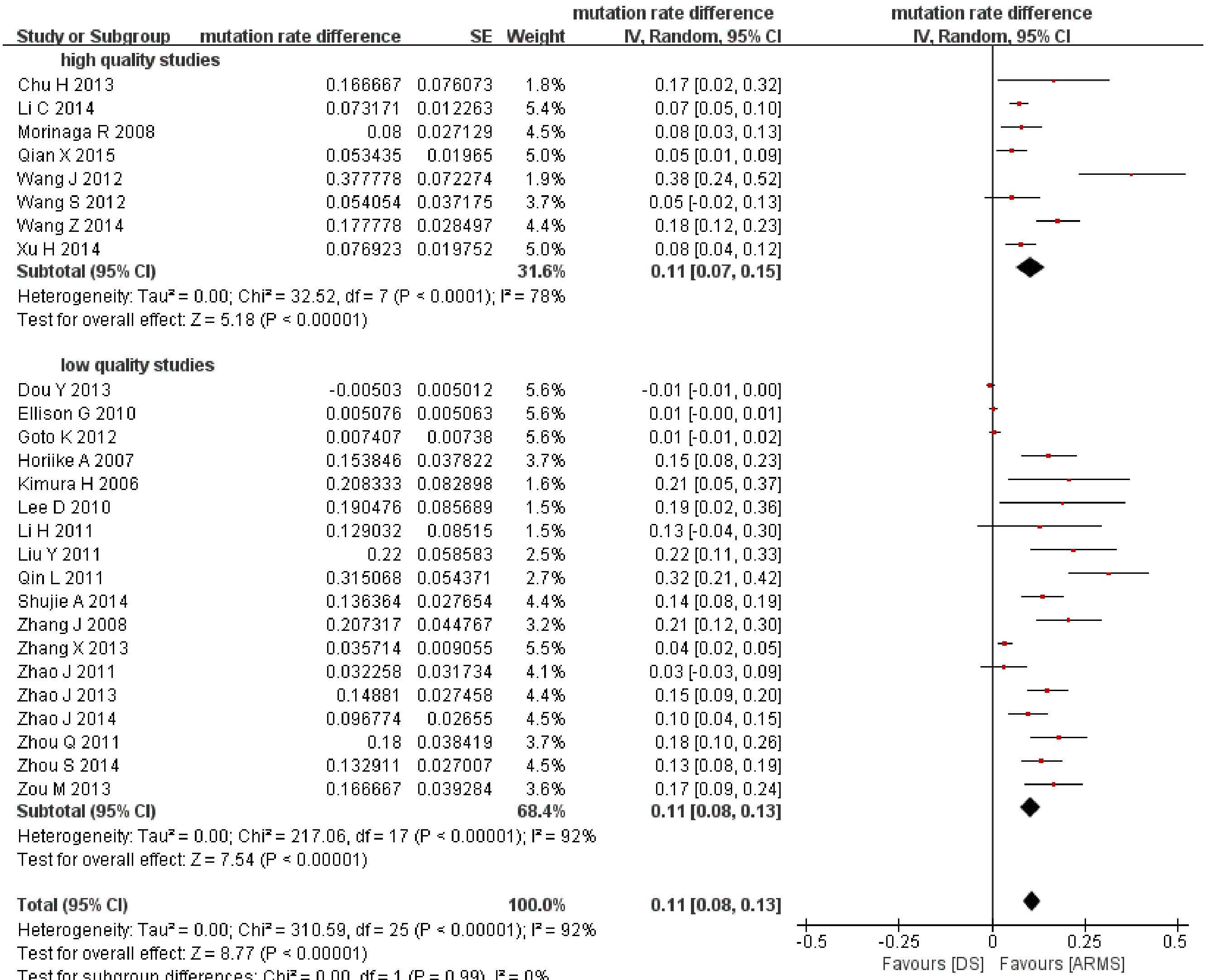

Supplementary Figure 2: Meta-analysis of mutation rate differences stratified by study quality.

Study or Subgroup	mutation rate difference	SE	Weight	mutation rate difference IV, Random, 95% CI
**non-Asian**				
Ellison G 2010	0.00507614	0.00506324	5.6%	0.01 [-0.00, 0.01]
**Subtotal (95% CI)**			**5.6%**	**0.01 [-0.00, 0.01]**
Heterogeneity: Not applicable				
Test for overall effect: Z = 1.00 (P = 0.32)				
**Asian**				
Chu H 2013	0.166667	0.076073	1.8%	0.17 [0.02, 0.32]
Dou Y 2013	-0.00503	0.005012	5.6%	-0.01 [-0.01, 0.00]
Goto K 2012	0.007407	0.00738	5.6%	0.01 [-0.01, 0.02]
Horiike A 2007	0.153846	0.037822	3.7%	0.15 [0.08, 0.23]
Kimura H 2006	0.208333	0.082898	1.6%	0.21 [0.05, 0.37]
Lee D 2010	0.190476	0.085689	1.5%	0.19 [0.02, 0.36]
Li C 2014	0.073171	0.012263	5.4%	0.07 [0.05, 0.10]
Li H 2011	0.129032	0.08515	1.5%	0.13 [-0.04, 0.30]
Liu Y 2011	0.22	0.058583	2.5%	0.22 [0.11, 0.33]
Morinaga R 2008	0.08	0.027129	4.5%	0.08 [0.03, 0.13]
Qian X 2015	0.053435	0.01965	5.0%	0.05 [0.01, 0.09]
Qin L 2011	0.315068	0.054371	2.7%	0.32 [0.21, 0.42]
Shujie A 2014	0.12987	0.027089	4.5%	0.13 [0.08, 0.18]
Wang J 2012	0.377778	0.072274	1.9%	0.38 [0.24, 0.52]
Wang S 2012	0.054054	0.037175	3.7%	0.05 [-0.02, 0.13]
Wang Z 2014	0.177778	0.028497	4.4%	0.18 [0.12, 0.23]
Xu H 2014	0.076923	0.019752	5.0%	0.08 [0.04, 0.12]
Zhang J 2008	0.207317	0.044767	3.2%	0.21 [0.12, 0.30]
Zhang X 2013	0.035714	0.009055	5.5%	0.04 [0.02, 0.05]
Zhao J 2011	0.032258	0.031734	4.1%	0.03 [-0.03, 0.09]
Zhao J 2013	0.14881	0.027458	4.4%	0.15 [0.09, 0.20]
Zhao J 2014	0.096774	0.02655	4.5%	0.10 [0.04, 0.15]
Zhou Q 2011	0.18	0.038419	3.7%	0.18 [0.10, 0.26]
Zhou S 2014	0.132911	0.027007	4.5%	0.13 [0.08, 0.19]
Zou M 2013	0.166667	0.039284	3.6%	0.17 [0.09, 0.24]
**Subtotal (95% CI)**			**94.4%**	**0.12 [0.09, 0.15]**
Heterogeneity: Tau² = 0.00; Chi² = 289.19, df = 24 (P < 0.00001); I² = 92%				
Test for overall effect: Z = 8.25 (P < 0.00001)				
**Total (95% CI)**			**100.0%**	**0.11 [0.08, 0.13]**
Heterogeneity: Tau² = 0.00; Chi² = 309.35, df = 25 (P < 0.00001); I² = 92%				
Test for overall effect: Z = 8.76 (P < 0.00001)				
Test for subgroup differences: Chi² = 55.35, df = 1 (P < 0.00001), I² = 98.2%				

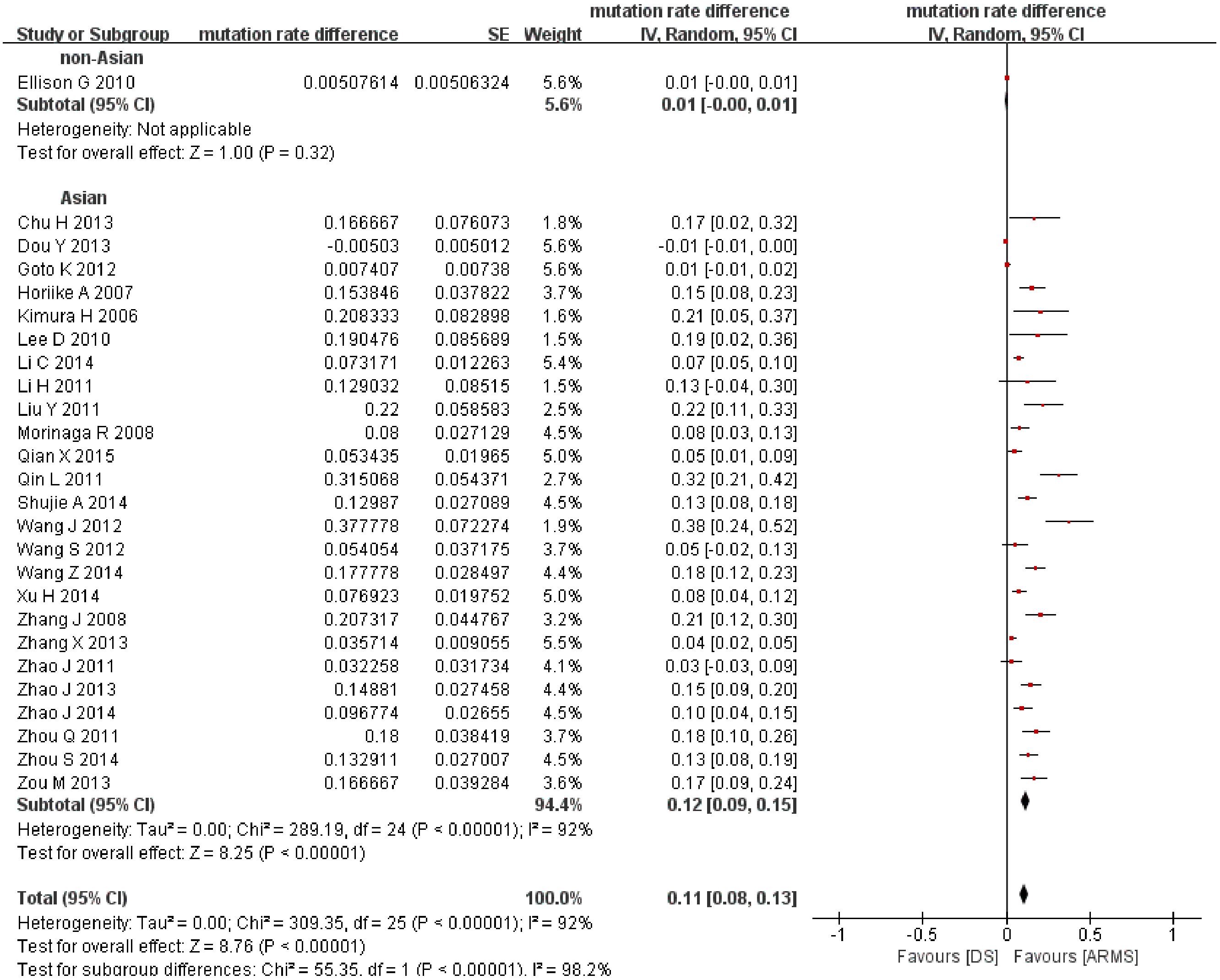

Supplementary Figure 3: Meta-analysis of mutation rate differences by ethnicity.

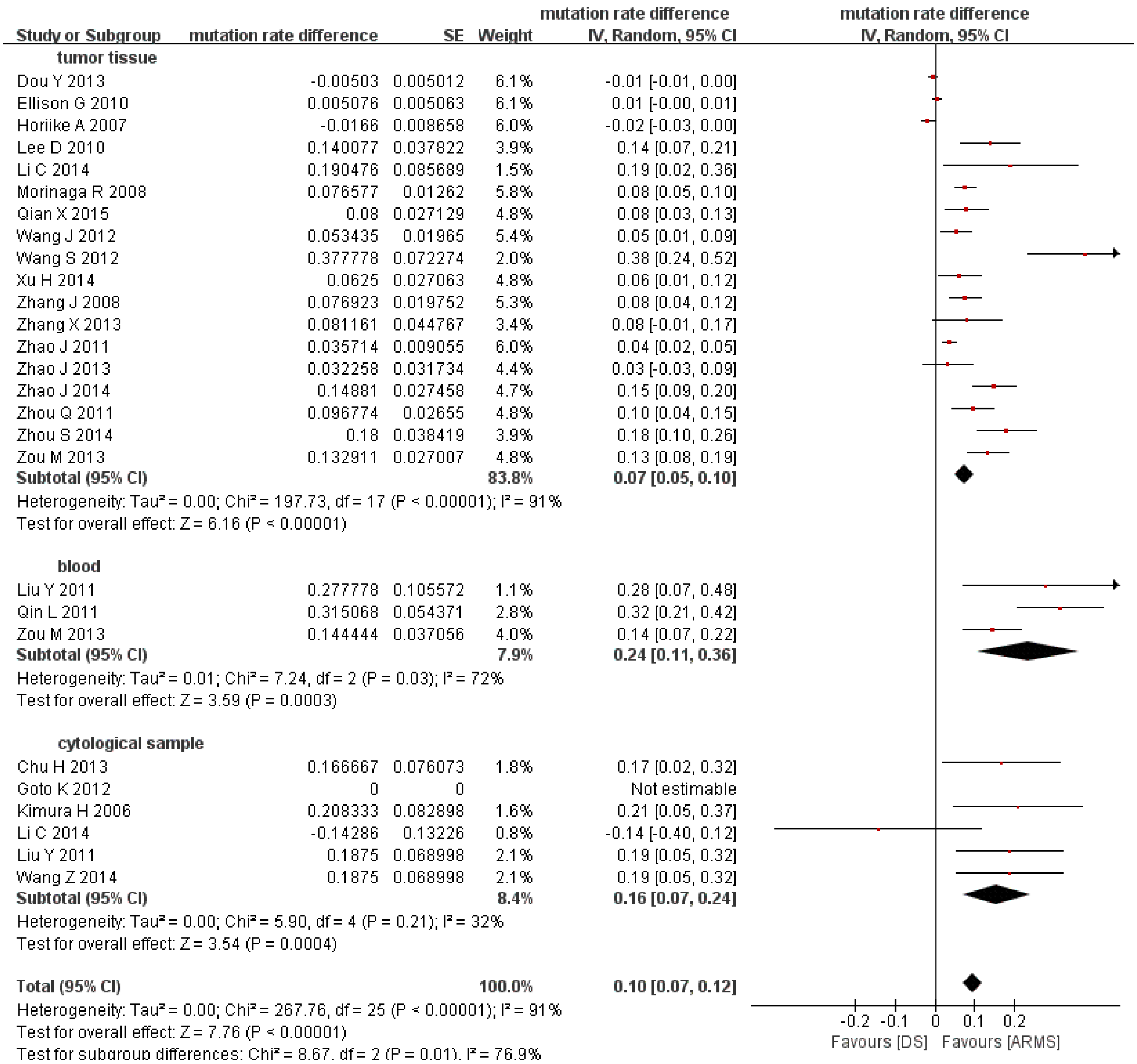

Supplementary Figure 4: Meta-analysis of mutation rate differences by specimen types.

For Supplementary Tables see in Suplementary Files
