# Supplementary material for: Comparison of direct sequencing and amplification refractory mutation system for detecting epidermal growth factor receptor mutation in non-small-cell lung cancer patients: a systematic review and meta-analysis
Source: Oncotarget. 2017 Jul 8;8(35):59552–62. doi: 10.18632/oncotarget.19110 (PMC5601754; doi:10.18632/oncotarget.19110)
Supplement: Supplementary file 2 [file oncotarget-08-59552-s002.docx]

| Supplementary Table 1: Quality assessment of cross-sectional study/part via AHRQ tool* | | | | | | | | | | |
| --- | --- | --- | --- | --- | --- | --- | --- | --- | --- | --- |
| Study id | Item 1 | Item 2 | Item 3 | Item 4 | Item 5 | Item 6 | Item 7 | Item 9 | Item 10 | Total |
| Chu H 2013 | 1 | 1 | 1 | 1 | 1 | 1 | 0 | 1 | 1 | 8 |
| Dou Y 2013 | 1 | 1 | 1 | 1 | 0 | 1 | 0 | 1 | 0 | 6 |
| Ellison G 2010 | 1 | 1 | 1 | 1 | 0 | 1 | 0 | 1 | 0 | 6 |
| Goto K 2012 | 1 | 1 | 1 | 1 | 0 | 0 | 0 | 1 | 0 | 5 |
| Horiike A 2007 | 1 | 1 | 0 | 1 | 0 | 1 | 0 | 1 | 1 | 6 |
| Kimura H 2006 | 1 | 1 | 0 | 1 | 0 | 1 | 0 | 1 | 1 | 6 |
| Lee D 2010** | NA | NA | NA | NA | NA | NA | NA | NA | NA | NA |
| Li C 2014 | 1 | 1 | 1 | 1 | 0 | 1 | 0 | 1 | 1 | 7 |
| Li H 2011** | NA | NA | NA | NA | NA | NA | NA | NA | NA | NA |
| Liu Y 2011 | 1 | 1 | 1 | 0 | 0 | 0 | 0 | 1 | 1 | 5 |
| Morinaga R 2008 | 1 | 1 | 1 | 1 | 0 | 1 | 0 | 1 | 1 | 7 |
| Qian X 2015 | 1 | 1 | 1 | 1 | 0 | 1 | 1 | 1 | 0 | 7 |
| Qin L 2011 | 1 | 1 | 1 | 0 | 1 | 0 | 0 | 1 | 1 | 6 |
| Shujie A 2014 | 1 | 1 | 1 | 1 | 0 | 0 | 0 | 1 | 1 | 6 |
| Wang J 2012 | 1 | 1 | 1 | 1 | 0 | 1 | 0 | 1 | 1 | 7 |
| Wang S 2012 | 1 | 1 | 1 | 1 | 0 | 0 | 1 | 1 | 1 | 7 |
| Wang X 2011 | 1 | 1 | 1 | 1 | 0 | 0 | 1 | 1 | 1 | 7 |
| Wang Z 2014 | 1 | 1 | 1 | 1 | 0 | 0 | 1 | 1 | 1 | 7 |
| Xu H 2014 | 1 | 1 | 1 | 1 | 1 | 0 | 1 | 1 | 1 | 8 |
| Zhang J 2008 | 1 | 1 | 1 | 1 | 0 | 0 | 0 | 1 | 1 | 6 |
| Zhang X 2013** | NA | NA | NA | NA | NA | NA | NA | NA | NA | NA |
| Zhao J 2011 | 1 | 0 | 0 | 0 | 1 | 0 | 1 | 1 | 1 | 5 |
| Zhao J 2013 | 1 | 1 | 1 | 1 | 0 | 0 | 0 | 1 | 1 | 6 |
| Zhao J 2014 | 1 | 1 | 0 | 0 | 0 | 0 | 0 | 1 | 1 | 4 |
| Zhou Q 2011 | 1 | 1 | 0 | 1 | 0 | 1 | 0 | 1 | 1 | 6 |
| Zhou S 2014 | 1 | 1 | 1 | 1 | 0 | 0 | 0 | 1 | 0 | 5 |
| Zou M 2013 | 1 | 1 | 1 | 0 | 0 | 1 | 0 | 1 | 1 | 6 |

*: AHRQ quality assessment tool includes 11 items covering data source, subject selection criteria, time period, subject selection method, blindness, quality assurance, exclusion for analysis, confounding, missing data handling, completeness of data collection and expected follow up. There are 3 options for each item, that is, yes, no and unclear. We assign 1 point if the answer is yes, otherwise 0. We excluded the items of confounding and expected follow up in this study, because they are irrelevant in prevalence study. Studies with total score >=7 are regarded as high quality.

**: these studies have only abstracts without full texts available, thus giving insufficient information for methodological quality assessment.

| Supplementary Table 2: Quality assessment of cohort study/part via NOS tool* | | | | | | | | | |
| --- | --- | --- | --- | --- | --- | --- | --- | --- | --- |
| Study id | Item 1 | Item 2 | Item 3 | Item 4 | Item 5 | Item 6 | Item 7 | Item 8 | Total |
| Zhao J 2013 | 1 | 1 | 1 | 1 | 2 | 1 | 1 | 1 | 9 |
| Wang X 2011 | 0 | 1 | 1 | 1 | 2 | 1 | 1 | 0 | 7 |
| Wang Z 2014 | 0 | 1 | 1 | 1 | 2 | 1 | 1 | 1 | 8 |
| Kimura H 2006 | 0 | 1 | 1 | 1 | 2 | 1 | 1 | 1 | 8 |
| Liu Y 2011 | 0 | 1 | 1 | 1 | 2 | 1 | 1 | 1 | 8 |
| Zhao J 2014 | 0 | 1 | 1 | 1 | 2 | 1 | 1 | 1 | 8 |
| Zhou Q 2011 | 0 | 1 | 1 | 1 | 2 | 1 | 1 | 1 | 8 |
| Zhou S 2014 | 0 | 1 | 1 | 1 | 2 | 1 | 1 | 1 | 8 |

*: NOS (Newcastle-Ottawa Scale) includes 8 items, that is representativeness, selection of non-exposed cohort, ascertainment of exposure, demonstration that outcome was not present at start of study, comparability of cohorts, outcome assessment, follow-up length, adequacy of follow up. Each satisfied item is assigned 1 point, while the item of comparability of cohort (item 5) can be given 2 points if fully satisfied. Studies with total score >=7 are regarded as high quality.
