# Supplementary material for: Comparison of direct sequencing and amplification refractory mutation system for detecting epidermal growth factor receptor mutation in non-small-cell lung cancer patients: a systematic review and meta-analysis
Source: Oncotarget. 2017 Jul 8;8(35):59552–62. doi: 10.18632/oncotarget.19110 (PMC5601754; doi:10.18632/oncotarget.19110)
Supplement: Supplementary file 3 [file oncotarget-08-59552-s003.docx]

Appendix 1: search strategy (with the example in MEDLINE via Ovid)

| 1. | Lung. ti,ab |
| --- | --- |
| 2. | Pulmonary. ti,ab |
| 3. | Cancer*. ti,ab |
| 4. | Carcinoma*. ti,ab |
| 5. | Adenocarcinoma*.ti,ab |
| 6. | Tumor*.ti,ab |
| 7. | Sequenc*.ti,ab |
| 8. | Amplification refractory mutation system.yi,ab |
| 9. | ARMS.ti,ab |
| 10. | Allele-specific polymerase chain reaction.ti,ab |
| 11. | Allele-specific PCR.ti,ab |
| 12. | PASA.ti,ab |
| 13. | ASP.ti,ab |
| 14. | 1 or 2 |
| 15. | 3 or 4 or 5 or 6 |
| 16. | 8 or 9 or 10 or 11 or 12 or 13 |
| 17. | 14 and 15 and 7 and 16 |
| 18. | Limit 17 to human |

The search strategy in EMBASE, CNKI and Wanfang were developed and adopted from this strategy.
